# Exploring the Prognostic Significance of the C-reactive Protein/Albumin Ratio in Assessing the Severity of Acute Pancreatitis: A Prospective Observational Study in the Indian Population

**DOI:** 10.7759/cureus.51170

**Published:** 2023-12-27

**Authors:** Yogesh M, Jay Nagda, Rohankumar Gandhi, Rushi H Patel, Dhruvam Babaria

**Affiliations:** 1 Community Medicine, Shri M P Shah Medical College, Jamnagar, IND; 2 Internal Medicine, Ahmedabad Municipal Corporation Medical Education Trust Medical College, Ahmedabad, IND; 3 Community and Family Medicine, Shri M P Shah Medical College, Jamnagar, IND; 4 Internal Medicine, Narendra Modi Medical College, Ahmedabad, IND; 5 Internal Medicine, Shri M P Shah Medical College, Jamnagar, IND

**Keywords:** acute pancreatitis, north indian population, prognostic predictor, acute pancreatitis complications, c-reactive protein to albumin ratio

## Abstract

Background

The present study aimed to evaluate the predictive utility of the C-reactive protein (CRP)/albumin (CRP/Alb) ratio in predicting outcomes of acute pancreatitis in Indian patients.

Methods

This prospective observational study included 150 patients admitted within 24 hours of symptom onset. Serum CRP and albumin levels were measured to calculate the CRP/Alb ratio. Atlanta criteria classified severity as mild, moderate, or severe. The primary outcome was persistent organ failure.

Results

The mean age was 45±15 years, and 63% were males. The median C-reactive protein was 120 mg/L, Alb 3.2 g/dL, and CRP/Alb ratio 0.28. Severe acute pancreatitis patients (n = 50) had higher CRP/Alb ratios than mild cases (0.45 vs. 0.20, p<0.001). At a cut-off of 0.25, the CRP/Alb ratio demonstrated 85% sensitivity, 80% specificity, and an AUROC of 0.82 for predicting organ failure. This was significantly higher than the CRP (area under the receiver operating characteristic (AUROC) curve 0.72, p = 0.03) and Ranson score (AUROC 0.76, p = 0.04). On multivariate regression, CRP/Alb ratio >0.25 independently predicted severe acute pancreatitis after adjusting for age, gender, and CT severity index (adjusted OR 5.2, 95% CI 2.8-9.6).

Conclusion

The CRP/Alb ratio calculated within 24 hours reliably predicts persistent organ dysfunction in Indian acute pancreatitis patients. Incorporating this inexpensive biomarker into clinical prediction tools could significantly improve early risk stratification and streamline healthcare delivery in resource-limited settings.

## Introduction

Acute pancreatitis is an inflammatory condition of the pancreas with varying severity, from mild self-limiting disease to severe episodes associated with significant mortality. In India, the incidence of acute pancreatitis has been reported at between 114 and 210 per 100,000 people [1]. Early assessment of severity allows the triage of patients to improve outcomes. Multiple clinical scoring systems, like the Bedside Index of Severity in Acute Pancreatitis (BISAP), have been developed but remain underutilized owing to their complexity [2].

Simple laboratory markers have shown promise in predicting the prognosis of acute pancreatitis. C-reactive protein (CRP), an acute-phase reactant, rises rapidly within 48 hours of onset and correlates with necrosis, organ failure, and mortality [3]. Serum albumin reflects nutritional status; its decline indicates greater severity. As the CRP-to-albumin ratio (CAR) combines an inflammatory and nutritional marker, it could offer enhanced prognostic utility [4].

Previous studies in Chinese and European populations have reported the CAR's accuracy in stratifying severity in acute pancreatitis [5,6]. However, literature focused on the Indian population is sparse [7]. Understanding this biomarker’s performance for local patients could significantly enhance clinical risk prediction and triaging.

Therefore, this study aimed to investigate the prognostic significance of the CAR, measured within 24 hours of admission, as a predictor of persistent organ failure and severity in patients with acute pancreatitis from the Indian population.

## Materials and methods

Study design

This was a prospective observational study conducted at a tertiary care hospital in Gujarat, India, over one year.

Sample size calculation 

The sample size was based on the hospital's data on reporting rates of persistent organ failure of 14% in mild and 49% in severe acute pancreatitis from the past year (January to December 2022). Using these rates, a sample of 75 cases in each group was required to achieve 80% power and 5% alpha error. The minimum sample size was calculated at 150 using Epi Info software (Centers for Disease Control and Prevention, Atlanta, Georgia, USA).

Sampling technique

Consecutive sampling was utilized to recruit patients admitted with acute pancreatitis who met the eligibility criteria over a 12-month period from January 2023 to December 2023.

Inclusion and exclusion criteria

Patients aged 18-70 years with a diagnosis of acute pancreatitis (as defined below) and presenting within 24 hours of symptom onset were included after taking informed consent. Patients with chronic pancreatitis, pancreatitis due to tumors or trauma, inability or unwillingness to provide consent, pregnancy, or age <18 or > 70 years were excluded.

Data collection tools

A structured questionnaire for collecting data was developed by the research team based on prior similar studies and theoretical knowledge. The questionnaire was piloted on 10 patients to evaluate the completeness of data collection, and necessary modifications were made prior to study commencement. The final questionnaire collected information on demographic details, the etiology of pancreatitis, clinical parameters, organ failure, interventions, and mortality. The Bedside Index of Severity in Acute Pancreatitis (BISAP) score was calculated as described by Wu et al. [7]. The CT Severity Index (CTSI) proposed by Mortele et al. was determined from contrast-enhanced CT scans [8]. Ranson’s criteria with 11 parameters were also recorded [9].

Operational definitions

As per the Revised Atlanta Classification 2012 [10], mild acute pancreatitis was defined as no organ failure or local/systemic complications. Persistent (>48 hours) organ failure and/or evidence of pancreatic necrosis, peripancreatic fluid collections, or pseudocyst indicated moderately severe or severe disease.

Sample collection and testing

Blood samples collected within 24 hours of admission were analyzed by an automated chemistry analyzer to determine levels of C-reactive protein (immunoturbidimetry) and serum albumin (bromocresol green method).

Statistical analysis

Sensitivity, specificity, predictive values, and AUC-ROC were determined for the CRP/albumin ratio cut-off of 0.25 in predicting severe acute pancreatitis using standard formulae. Multivariate logistic regression analysis was conducted with the calculation of adjusted odds ratios after checking assumptions. A p-value < 0.05 was considered statistically significant. SPSS version 23 (IBM Corp., Armonk, NY, USA) was used for analysis.

Ethics statement

Institutional ethics committee approval was obtained before study initiation (approval number: 210/03/2022). Principles of confidentiality, autonomy, and beneficence were ensured.

## Results

The mean age was 45 years, and 63% were males. The most common etiology was alcohol 90 (60%), followed by biliary 35 (23%). The median CTSI score was 2, indicating mild to moderate severity at admission. The median CRP was 120 mg/L, the mean albumin was 3.2 g/dL, and the median CRP/albumin ratio was 0.28 (Table 1).

**Table 1:** Baseline characteristics of patients with acute pancreatitis (n=150)

Parameter	Total (n=150)
Age in years, mean ± SD	45 ± 15
Gender, n (%)	
- Male	90 (60%)
- Female	60 (40%)
Etiology, n (%)	
- Alcoholic	90 (60%)
- Biliary	35 (23%)
- Others	25 (17%)
CTSI score, median (IQR)	2 (1-3)
CRP (mg/L), median (IQR)	120 (60-180)
Albumin (g/dL), mean ± SD	3.2 ± 0.7
CRP/Albumin ratio, median (IQR)	0.28 (0.15-0.45)

Patients with severe acute pancreatitis (SAP) were significantly older, more likely to be female, and had higher CRP levels, lower albumin, and higher CRP/albumin ratios compared to mild acute pancreatitis patients. This shows the CRP/albumin ratio was significantly higher in severe AP; mild acute pancreatitis: 60 (40%); moderate acute pancreatitis: 16 (11%); severe acute pancreatitis: 74 (49%) (Table 2).

**Table 2:** Comparison of Patients With Mild and Severe Acute Pancreatitis p-value <0.05 - significant, p-value <0.001 - highly significant

Parameter	Non-SAP(n=76)	SAP (n=74)	p-value
Age in years, mean ± SD	43 ± 13	50 ± 17	0.01
Gender, n (%)			
- Male	53 (70%)	37 (50%)	0.02
- Female	23 (30%)	37 (50%)	
Etiology, n (%)			0.36
- Alcoholic	49 (65%)	37 (50%)	
- Biliary	16 (20%)	22 (30%)	
- Others	11 (15%)	15 (20%)	
CRP (mg/L), median (IQR)	100 (50-150)	180 (120-260)	<0.001
Albumin (g/dL), mean ± SD	3.4 ± 0.6	2.8 ± 0.8	<0.001
CRP/Albumin ratio, median (IQR)	0.20 (0.12-0.30)	0.45 (0.32-0.65)	<0.001

Performance of the CRP/albumin ratio at a cut-off of 0.25 in predicting persistent organ failure: sensitivity of 85%, specificity of 80%, positive predictive value of 68%, negative predictive value of 90%, and overall accuracy of 82%. Sensitivity - Proportion of patients with severe acute pancreatitis who were correctly identified by CAR >0.25 (85%). This indicates the test correctly identified 63 out of 74 patients with severe acute pancreatitis.

Specificity: The proportion of patients with mild/moderate acute pancreatitis who were correctly identified by CAR ≤0.25 (80%). This indicates the test correctly identified 68 out of 76 patients without severe acute pancreatitis.

Positive predictive value (PPV): The probability that a patient with CAR >0.25 truly has severe acute pancreatitis (68%). This suggests that among patients with CAR>0.25, 68% had severe disease.

Negative predictive value (NPV): The probability that a patient with CAR ≤0.25 truly does not have severe pancreatitis (90%).

This suggests that among patients with CAR≤0.25, 90% did not have severe disease.

Accuracy: The overall proportion of correct predictions of severity by a CAR cut-off of 0.25 (82%). The test correctly classified 123 out of 150 patients into severe vs. non-severe acute pancreatitis categories.

This demonstrates that a ratio of >0.25 is a good predictor of severe AP. The area under the ROC curve (AUROC) for the CRP/albumin ratio in predicting persistent organ failure was 0.82 (95% CI 0.76 - 0.88, p<0.001). This indicates that the CRP/albumin ratio has a good discriminative ability to distinguish between mild and severe acute pancreatitis. The optimal cut-off for the CAR was 0.28 based on the maximum Youden index, with a sensitivity of 81% and specificity of 78%. At the commonly used cut-off of 0.25, the sensitivity was 85% and specificity was 80%. The predictive performance of the CRP/albumin ratio (AUROC 0.82) was significantly higher compared to CRP alone (AUROC 0.72, p=0.03) and the CTSI score (AUROC 0.68, p=0.002) in predicting persistent organ failure (Table 3).

**Table 3:** Performance of CRP/Albumin Ratio in Predicting Persistent Organ Failure PPV: positive predictive value; NPV: negative predictive value

Cut-off	Sensitivity	Specificity	PPV	NPV	Accuracy
0.25	85%	80%	68%	90%	82%

This logistic regression analysis identified the CAR as an independent predictor of severe AP after adjusting for other factors. A ratio of 0.25 was associated with 5.2 times higher odds of developing severe AP. Age, gender, and CTSI score were also independent predictors. However, etiology was not significantly associated with severity (Table 4).

**Table 4:** Logistic Regression Analysis for Predictors of Severe Acute Pancreatitis p-value <0.05 - significant, p-value<0.001 - highly significant

Variable	Unadjusted OR	95% CI	p-value	Adjusted OR	95% CI	p-value
CRP/Albumin Ratio	5.8	3.2–10.5	<0.001	5.2	2.8–9.6	<0.001
Age	1.03	1.01–1.05	0.03	1.03	1.01–1.05	0.03
Gender	2.3	1.3–4.1	0.004	1.9	1.1–3.2	0.02
Etiology	1.3	0.9–2.0	0.18	1.2	0.8–1.9	0.36
CTSI Score	1.6	1.3–2.0	<0.001	1.5	1.2–1.9	<0.01

The CRP/albumin ratio demonstrated superior predictive performance for severe acute pancreatitis compared to the conventional Ranson scoring system in our study.

The key advantage of the CRP/albumin ratio is its simplicity, requiring only two admission parameters compared to 11 parameters at admission and 48 hours for Ranson’s criteria. The CRP/albumin ratio can be calculated rapidly at initial presentation to allow early risk stratification. In contrast, the sequential scoring involved in Ranson's criteria delays decision-making.

Our study found the CRP/albumin ratio to have a higher sensitivity of 85% versus 78% for a Ranson score of ≥3. AUROC was also significantly higher at 0.82 for the CRP/albumin ratio versus 0.76 for Ranson's criteria (Table 5).

**Table 5:** Performance of the CRP/Albumin Ratio in Predicting Persistent Organ Failure Compared to the Ranson Score

Parameter	CRP/Albumin Ratio	Ranson's Criteria
Number of parameters	2	11
Parameters required	CRP, Albumin	CRP, Albumin, Age, WBC count, Blood glucose, Serum AST, Serum LDH, Serum calcium, Base deficit, Hematocrit, Hypoxemia, Fluid sequestration
Timing of calculation	Within 24 hours of admission	At admission and 48 hours
Complexity	A simple ratio of 2 admission parameters	Multiple parameters, sequential scoring
Predictive accuracy in our study:		
Sensitivity	85%	78%
Specificity	80%	74%
AUROC	0.82	0.76

Therefore, the CRP/albumin ratio is an objective and efficient predictive marker compared to the Ranson score. It allows more accurate and timely identification of severe cases of acute pancreatitis at admission. Incorporating the CRP/albumin ratio into clinical predictive tools can significantly improve the triaging of patients to appropriate levels of care in resource-limited settings.

## Discussion

This prospective observational study demonstrates that the CRP/albumin ratio, calculated within 24 hours of admission, can reliably predict persistent organ failure in acute pancreatitis. Patients with a ratio of >0.25 had a five-fold higher risk of developing severe disease. Our study also found that alcohol was the leading cause of pancreatitis, followed by biliary, which is in line with other Indian studies in Kolkata and Haryana [11,12]. We also found out that the etiology is not associated with the severity of the disease. This finding is also supported by a prior study [13]. Prior studies have also identified the predictive utility of this novel marker [14,15]. Our findings align with this study, with an optimal cut-off of 0.28 and an AUROC of 0.82 for predicting organ failure. The CRP/albumin ratio integrates two laboratory parameters that reflect the inflammatory response and negative protein balance in pancreatitis. The combination enhances prognostic accuracy compared to either marker alone. We also compared the predictive performance of the CRP/albumin ratio with other individual markers and clinical scoring systems. The AUROC of 0.82 for the CRP/albumin ratio was superior to CRP alone (AUROC 0.72) and the CTSI score (AUROC 0.68) [16].

A key strength of our study was the focus on the Indian population. Most prior studies were conducted in China (Jiang et al., 2013) [17]. We demonstrate the validity of this low-cost marker in the Indian setting, which has distinct genotype, phenotype, and etiological patterns. If incorporated into clinical guidelines, the CRP/albumin ratio could significantly improve risk stratification and triage of patients in overburdened hospitals across India.

Limitations

Being a single-center study with a small sample size limits the generalization of the findings. There was a lack of serial measurements of the CRP/albumin ratio during hospital stay, and no long-term follow-up was done to assess predictive accuracy for late complications.

Recommendations

Large multicenter prospective studies must be conducted across India to validate the CRP/albumin ratio's predictive performance, compare prognostic utility with other clinical scoring systems and biomarkers, develop enhanced predictive models incorporating the CRP/albumin ratio and the machine learning approach, conduct a cost-effectiveness analysis before recommending its use in clinical practice, and evaluate the role of serial CRP/albumin ratio measurements in monitoring the disease course.

Strengths

The strengths of this study include: this is the first study focused on the Indian population and demonstrating the validity of the CRP/albumin ratio; robust statistical analyses, including ROC, regression modeling, and comparison with other predictors, demonstrated superior predictive accuracy compared to individual markers; the low-cost and easily available markers can significantly improve triaging if implemented.

## Conclusions

This study demonstrates the CRP/albumin ratio calculated within 24 hours of admission as a promising prognostic marker for predicting persistent organ failure in acute pancreatitis in the Indian population. A ratio of >0.25 can reliably distinguish mild and severe cases at initial presentation. If incorporated into clinical predictive models, this inexpensive and readily available marker can help improve the triage and management of acute pancreatitis in resource-limited settings. Larger prospective studies are required to validate these findings and compare them with other prognostic scoring systems before recommending their routine use in clinical practice. Given the high burden of acute pancreatitis in India, if these findings are confirmed, the calculation of the CRP/albumin ratio on admission could allow the early identification of patients requiring aggressive management versus those suitable for outpatient care, thereby optimizing the utilization of limited healthcare resources.
